# Factors Associated with Employment in a Cohort of Patients with Systemic Sclerosis

**DOI:** 10.3390/jcm14134764

**Published:** 2025-07-05

**Authors:** Cristina A. Vrancianu, Cristiana Grigore, Ioan Ancuta, Mihai Bojinca, Ana Maria Gheorghiu

**Affiliations:** 1 Faculty of Medicine, Carol Davila University of Medicine and Pharmacy, 050474 Bucharest, Romania; 2 Internal Medicine and Rheumatology Department, Cantacuzino Clinical Hospital, 020475 Bucharest, Romania; 3 Internal Medicine Department, Sf. Ioan Hospital, 042122 Bucharest, Romania

**Keywords:** systemic sclerosis, employment status, early retirement

## Abstract

**Background/Objectives:** Systemic sclerosis (SSc) is a multisystemic chronic autoimmune disease, which leads to disability and possibly early retirement. The objective of our study was to explore the associations between employment status (ES) and demographic, clinical and functional features in a single-center EUSTAR cohort. **Methods:** Consecutive patients with SSc examined between November 2011 and June 2023, who were under the age of retirement in our country (62 years for women, 65 for men at the time), were included. All patients underwent a comprehensive clinical assessment and filled in a work assessment questionnaire as well as two validated health-related questionnaires: the Scleroderma Health Assessment Questionnaire (SHAQ) and the Duruoz Hand Index (DHI). Associations between ES and potential predictors (education level, disease characteristics, work conditions, SHAQ and DHI) were tested using logistic regression adjusted for age and gender. **Results:** Ninety-one patients (mean ± SD age 53.7 ± 11.8 years, twenty-two with diffuse skin involvement, fifty-six with a history of digital of digital ulcers (DUs)), were included. Only 22 patients were still employed, while 69 were retired, of which 38 retired because of SSc. Among the employed, nine performed manual labor, nine spent many hours standing and three had to work in a cold environment. When potential predictors were tested separately, adjusted for age and sex, patients with higher education (OR (95% CI) 11.36 (2.03–63.36), *p* = 0.006) and no history of digital ulcers had higher odds of being employed. The presence of joint contractures and weightlifting as a work demand were associated with unemployment. In a multivariable model, higher education (OR 5.91, 95% CI 0.97–36.09, *p* = 0.054 and younger age (OR 0.90, 95% CI 0.85–0.96, *p* = 0.001) were independently associated with continued employment. High school education did not show a significant effect (OR 0.089, 95% CI 0.015–0.530, *p* = 0.008). Patients with a history of digital ulcers had the lowest employment rates compared to those with no digital ulcer history. No significant associations were found between employment status and SHAQ or DHI scores. **Conclusions:** SSc is associated with significant work disability and early retirement. Higher education, the lack of Dus and younger age were highly associated with staying employed. Given the rarity of SSc, we consider that our good sample size *(n* = 91) reflects disease prevalence, but results should be tested in other studies and the single center should be considered when interpreting generalizability.

## 1. Introduction

The ability to work represents an important part in adult life, because it provides income, creates possibilities for social interactions and it also contributes to socioeconomic development [1,2,3]; it has multiple determinants, from work environment (cold temperatures, physical job) to individuals’ characteristics [4,5]. Everyday chores and occupations maintain an equilibrium in what concerns activities of daily life (ADL) [6]. It has also been proven that depression in people with limitations in work capacity is more common than in the general population [7]. Consequently, physical disability has a significant impact on the individual from multiple perspectives. Systemic sclerosis (SSc) is a chronic autoimmune disease characterized by microvascular injury and extensive fibrosis of the skin and internal organs [8]. Clinical manifestations such as digital ulcers (DU), reduced hand mobility as well as fatigability [5,9,10] have a major impact in SSc patients’ lives. Quite often, they are forced to change their jobs or to call on early retirement. According to recent systematic reviews and studied multicenter cohorts, including data from EUSTAR and GENISOS registries, more than 50% of patients with SSc experience work disability within the first year of diagnosis, with a significant number calling on early retirement [1,11]. Several studies regarding work disability in other rheumatic diseases such as ankylosing spondylitis (AS) and rheumatoid arthritis (RA) are available [12,13,14], but the heterogeneous traits of SSc make it more difficult to approach. Data on predictors of employment loss in SSc remain inconsistent; therefore, there is a need for more comprehensive research to understand the causes that lead to work disability. The aim of this study is to assess employment status, including early retirement (ER), and the associations of ES with demographic and disease characteristics and with patients’ health-related questionnaires.

## 2. Materials and Methods

Consecutive patients with SSc examined in our center in the period December 2011–June 2023 who were under the age of retirement in our country (62 years for women and 65 for men at the time) were included. Patients had a full SSc assessment and filled in a work assessment questionnaire and two other health-related questionnaires. All patients gave written informed consent. This study was approved by the local ethics committee of Cantacuzino Clinical Hospital.

Demographic variables included age, sex, area of residence (urban or rural), marital status and professional status (professionally active or retired). Patients also reported if they had children under 18 years old who needed their care. Education level was initially divided into “lower” (primary, secondary, vocational) and “higher” (high school or university). However, for finer stratification and comparability, we subsequently reclassified education into three distinct categories: low (primary/vocational), high school and university level, and used this classification in the final multivariable analysis.

Patients were also asked to report their last occupation, which was defined as their last job that could provide an income, and if they needed to commute in order to arrive at their workplace or not. The work assessment questionnaire covered the following: current and last occupation, workplace environment and demands; type of work (manual or not), posture (prolonged orthostatism), neurophysiological effort, remote work, gesture, weightlifting, microclimate (cold, humidity, draft) and work schedule (8 h/day, 8 h/2 rounds, 8 h/3 rounds). Patients were also asked whether they had retired due to disease-related reasons. Although this questionnaire is not formally validated, its structure was consistent with occupational health surveys used in similar studies.

Other variables were seniority, work capacity (number of days of sick leave in the last 12 months, degree of disability) and early retirement because of the disease.

Disease-related variables were represented by cutaneous subsets of disease (limited cutaneous (lSSc) and diffuse cutaneous (dSSc), disease duration, history of arthritis, history of digital ulcers or myositis, joint contractures and the modified Rodnan skin score (mRSS), as well as antibodies, inflammatory syndrome, heart ultrasound and pulmonary evaluation. Subgroup analysis was performed based on cutaneous disease subtype (limited vs. diffuse). Employment rates and associations were explored separately in each group using stratified descriptive and logistic analyses.

Patients completed two additional validated self-administered questionnaires: Scleroderma Health Assessment Questionnaire (SHAQ) and Duruoz hand index (DHI). The SHAQ and DHI questionnaires were administered in their validated Romanian-language versions during the same clinical visit as the full SSc assessment. SHAQ evaluates global disability and includes eight categories of questions that form the HAQ-Disability Index and five visual analog-scales (VAS), with ranges from 0 (no disability) to 3 (high disability) [15,16]. DHI assesses hand function and has a range from 0 to 90 [17,18]. Completion was supervised by study personnel to ensure clarity and accuracy, and patients filled out the forms independently.

Statistical analysis was performed in IBM SPSS Statistics 22 program [19]. The associations between ES and potential predictors, such as education, socio-economic status, disease features and health-related questionnaires, were tested using logistic regression adjusted for age and gender. For all analyses, *p* < 0.05 was considered statistically significant.

## 3. Results

Demographic and disease characteristics at baseline are presented in Table 1. In short, there were 91 patients included, of which eight were male (8.8%) with a mean ±SD age of 53.7 ± 11.8 years. Sixty-three patients (69.2%) lived in urban areas, while twenty-five (27.5%) lived in rural areas.

Regarding the level of education, our results showed that 18.7% (17 cases) completed lower secondary level and 22% (20 cases) vocational school, while 33% (30 cases) completed high school and 23 patients (25.3%) completed a higher education level (university). Overall, 53 patients were considered to have a high level of education, defined as completing high school and/or university. Twenty-two patients were professionally active, while a large number (sixty-nine out of ninety-one) were retired, thirty-eight of whom because of the disability determined by the disease. Of those active professionally, 9 (40.9%) had to do manual labor, 9 (40.9%) had to spend many hours at work standing, 3 (13.6%) had a cold and 1 (4.5%) a moist work environment (Table 2).

When potential predictors were tested separately in logistic regression models adjusted for age and sex, higher education (OR (95% CI) 11.36 (2.03–63.36, *p* = 0.006) had significantly increased odds of being professionally active. More specifically, they were over 11 times more likely to be employed compared to those with lower education. A positive history of digital ulcers (OR (95% CI) 0.19 (0.05–0.7, *p* = 0.012) was also a significant negative predictor. Patients who had experienced digital ulcers had a 81% reduction in the odds of being professionally active. Joint contractures (OR (95% CI) 0.22 (0.05–0.95, *p* = 0.043) were also associated significantly with decreased odds of being professionally active. The subset of disease at diagnosis had no influence on employment status, nor did an increased mRSS score. The only work-related variable that was associated with unemployment was heavy lifting (OR (95% CI) 0.19 (0.04–0.95, *p* = 0.044).

No significant associations were observed for manual labor, pulmonary function tests (FVC), left ventricular ejection fraction (LVEF), SHAQ or DHI scores and some of them were not retained in the final multivariate analysis. These results are presented in Table 3.

We also performed a multivariable logistic regression including age, sex, disease subset at diagnosis, history of digital ulcers, joint contractures, SHAQ score, mRSS, work demands (weightlifting) and education level.

When education was analyzed as a dichotomous variable (higher vs. lower education), higher education was strongly associated with continued employment (OR (95% CI) 5.91, (0.97–36.09), *p* = 0.054) in the multivariate model adjusted for age and relevant clinical covariates. To explore this association further, we divided education into three categories (low= primary/vocational, high school, and university. Compared to patients with low education, high school education was unexpectedly associated with lower odds of being professionally active (OR (95% CI) 0.089, (0.015–0.530), *p* = 0.008. University education, however, was not significantly associated with employment status (OR (95% CI) 0.539, (0.160–1.816), *p* = 0.318).

Other independent predictors of employment in the multivariable logistic regression model included younger age (OR (95% CI) 0.90 (0.84–0.97), *p* = 0.007, while digital ulcers or joint contractures did not influence employment. (Table 4).

These findings suggest that traditional clinical severity indices and general workload indicators may not fully capture the occupational impact of systemic sclerosis.

## 4. Discussion

Professional life plays an important role for every adult in contemporary society. In this study, we found work disability and early retirement due to systemic sclerosis to be significant and we showed that education can influence whether the patient remains professionally active or calls on early retirement

Recent systematic reviews and cohort studies have underscored the wide range of employment rates reported in SSc, varying from 25% to over 60%, depending on disease duration, severity and healthcare contexts [1]. These findings reflect the multifactorial nature of work disability in SSc, which is not only represented by physical impairment, but also by disease-specific symptoms (e.g., digital ulcers, Raynaud’s), fatigue, cognitive burden and quality of life indicators. Our results are consistent with this complexity, showing that functional indices such as SHAQ and DHI may not fully capture the occupational consequences of SSc.

In our study, a higher level of education, defined as completing high school or university, was associated with a higher level of employment and professional activity. This confirms previous studies, such as a large study on the GENISOS cohort (Genetic versus Environment In Scleroderma Outcome Study), conducted in 2011 in the United States [11], which showed that a lower level of education is associated with early retirement; they concluded that patients with lower educational level are expected to occupy positions that are physically demanding [11]. Moreover, a study which evaluated patients with scleroderma from Belgium, showed similar results with patients with work disability being less highly educated [20]. Previous analyses grouped high school and university education together, but our revised model revealed that high school education, when assessed separately, was unexpectedly associated with lower odds of remaining professionally active. This finding may reflect unmeasured differences in occupational demands, work stability or socioeconomic background among individuals with high school education. In contrast, university education did not show a statistically significant effect, possibly due to the limited sample size. These findings suggest a more nuanced role of education in shaping employment outcomes in systemic sclerosis and underscore the need for larger, prospective studies. We have gathered detailed information regarding labor conditions, and we showed that not having lifted weights is associated with staying employed but, interestingly, manual labor did not influence employment in our study. This may be perhaps because patients with higher education also have a higher income, which in turn leads to a better health-related quality of life [21]; moreover, they perform more mentally demanding work, which subsequently has a positive influence on cognitive status later on [22].

Our results also showed that patients who never had DUs tend to stay employed longer and have lower work disability compared to patients with a positive history of digital ulcers. A report from the DUO cohort [23] where DU occurrence was divided into four categories (no-DU, episodic, recurrent and chronic DUs) found the highest work impairment in patients with chronic DUs. Another study showed that severe vasculopathy, as well as renal involvement and FVC, were significantly associated with unemployment status in a univariate analysis; however, they did not remain statistically significant in the multivariable analysis after adjustment for disease duration, age and sex [24]. Other cohorts [5,15] found no association between DUs and work disability, but those cohorts had lower proportions of patients with DUs than in our study. Moreover, there was no data available regarding the occurrence manner of DUs in our cohort.

Our final multivariable model confirmed that higher education and younger age are independently associated with remaining professionally active in patients with systemic sclerosis, while digital ulcers and joint contractures did not demonstrate statistical significance. Contrary to expectations, self-reported functional disability (SHAQ) and cutaneous involvement (modified Rodnan skin score) did not reach statistical significance, which may reflect adaptation strategies or the influence of education level on perceived disability.

Several studies have shown that disease subset also has an influence on employment status, but this was not observed in our study. Hudson et al. provided evidence that work disability in SSc is associated with diffuse cutaneous involvement in a study on 643 patiens [1]. However, in another study performed on women with limited cutaneous SSc, Sandqvist et al. showed that although 50% of the cohort had a working disability, better working ability was associated with worse skin scores compared to women with lower working ability [2]. In our study, mRSS did not show any association with employment status.

Interestingly, no significant associations were found between employment status and several clinical or functional measures, including modified FVC, LVEF, SHAQ, or Duruoz Hand Index (DHI) in either adjusted or multivariable models. This lack of association may be due to limited statistical power in our study, but it may also point to a dissociation between physical disability and work ability. These tools, while validated for measuring disease activity and function, may not fully reflect the cognitive, psychosocial, and environmental dimensions that influence occupational participation. It is also possible that patients adapt their roles or modify their work environments, compensating for clinical limitations in ways not captured by current instruments. Future studies should include multidimensional tools that evaluate both physical limitations and workplace context. Joint contractures were independently associated with reduced likelihood of being professionally active. Functional impairment caused by contractures likely contributes to early work disability and socioeconomic burden. These results emphasize the importance of early intervention of rehabilitation programs which target mobility preservation. The association between joint contractures and work capacity would be worth studying further on larger cohorts, especially since it is currently disputed in other studies [25].

Other clinical manifestations like history of arthritis or myositis, as well as laboratory findings, had no impact on early retirement in our study.

Public data regarding the retirement from work due to disability as of 01.2023 show that out of 753 retired persons with first degree of invalidity, 632 are women, of the 939 with second degree of invalidity, 838 are women, and of the 852 with third degree of invalidity, 748 are women. Of the 91 patients in this study, the vast majority were females (83), which would make a statistical analysis regarding early retirement according to gender less reliable. Moreover, we found no publicly available data related to early retirement in patients with rheumatological diseases, and even less regarding patients with systemic sclerosis [25].

In light of these findings, workplace adaptations—such as ergonomic interventions, reduced physical strain and flexible scheduling—have become increasingly important for employment retention in SSc. Psychosocial support, including occupational counseling and social reintegration programs, may also play a key role. These aspects are often underreported in research and not utilized in practice. Policymakers and healthcare providers should consider integrative strategies that go beyond disease management and address work sustainability.

Our study has several limitations, such as the relatively low number of patients, and the lack of publicly available data for early retirement in the general population. Moreover, the evaluation of SSc patients by questionnaires, anamnesis and clinical and laboratory examinations may not be enough. An approach where patients present from their personal perspective the meaning of work transitions, as well as their potential causes, may be useful.

One limitation of this study is the absence of an external control group. Without comparison to other groups of patients with other chronic diseases (e.g., rheumatoid arthritis, ankylosing spondylitis) or the general working-age population, it is difficult to determine whether the observed unemployment and early retirement rates are uniquely driven by systemic sclerosis or reflect broader patterns of chronic illness. Although our analysis focuses on internal predictors within the SSc cohort, future comparative studies are warranted to better contextualize these findings.

An additional limitation is the lack of temporal stratification over the 12-year inclusion period (2011–2023). Changes in medical management, occupational health regulations and economic factors during this time may have influenced employment status. However, the sample size and retrospective design did not allow for a reliable time-based subgroup analysis. Future studies should explore the impact of temporal trends on employment outcomes in systemic sclerosis.

Another important limitation is the retrospective nature of data collection for occupational variables. Information regarding job history, physical demands and environmental conditions (e.g., cold exposure, manual labor) was based entirely on patient self-reporting. Since many patients had retired several years before inclusion in the study, there is a substantial risk of recall bias and subjective interpretation. This may have led to underestimation or overestimation of certain work-related factors.

Another limitation is represented by the assessment of digital ulcers, which was limited to presence or absence of DU, without further stratification into episodic, recurrent or chronic patterns. These limitations reduce the ability to draw a significant conclusion. However, the significant association supports existing evidence related to presence of DU and hand function impairment which leads to disability and lower ability to work.

Future studies will need a greater number of patients, as well as a follow-up, in order to assess their clinical and professional evolution along with the association between the two.

## 5. Conclusions

In conclusion, SSc is associated with substantial work disability and unemployment. In our cohort, remaining professionally active was associated with higher education, absence of digital ulcers and lack of lifting weights at work. In contrast, commonly used clinical and functional indices (SHAQ, DHI, FVC) were not predictive of employment status. These findings highlight the multifactorial nature of work disability in systemic sclerosis and emphasize the importance of education, vascular complications and workplace physical demands.

## Figures and Tables

**Table 1:** Clinical and demographic characteristics of the patients.

Characteristics	N (%)
Age, years	53.7 (11.8)
Male gender	8 (8.8)
dcSSc	22 (24.2)
History of Digital ulcers	56 (61.5)
Anti-Scl70 Antibodies positive	40 (44)
Joint contractures	34 (37.4)
Urban environment	63 (69.2)
**Education**	
Elementary and secondary	17 (18.7)
Vocational school	20 (22)
High school	30 (33)
University	23 (25.3)
Retired	69 (75.8)
SSc-related retirement	38 (41.8)

**Table 2:** Work-related characteristics of the patients.

	Active Professionally, N (%)	Retired, (N%)
Higher education	20 (90.9)	33 (47.8)
Manual labor	9(40.9)	37 (53.6)
Heavy lifting	2 (9.1)	26 (37.7)
Neuropsychological effort	14 (63.6)	43 (62.3)
Remote work	5 (22.7)	8 (11.6)
Cold work environment	3 (13.6)	28 (40.6)
Moist work environment	1 (4.5)	27 (39.1)
Work standing for many hours	9 (40.9)	41 (59.4)

**Table 3:** Associations between employment status and clinical, functional, and work-related characteristics. Logistic regression adjusted for age and sex.

Predictor	OR (95% CI)	*p* Value
**Higher education**	**11.36 (2.03–63.36)**	**0.006**
**Heavy lifting**	**0.19 (0.04–0.95)**	**0.044**
Manual labor	0.56 (0.19–1.61)	0.281
dcSSc	4.38 (0.99–19.47)	0.052
**DU History**	**0.19 (0.05–0.70)**	**0.012**
**Joint contractures**	**0.27 (0.08–0.88)**	**0.030**
mRSS	0.090 (0.79–1.01)	0.091
SHAQ score	0.99 (0.98–1.01)	0.512
DHI score	0.56 (0.30–1.05)	0.07
FVC	1.03 (0.99–1.08)	0.175
LVEF	1.10 (0.85–1.41)	0.475

**Table 4:** Multivariate logistic regression analysis of predictors of employment status in patients with systemic sclerosis. Odds ratios (OR) adjusted for age.

	R (95% CI)	*p*
**Higher education**	**5.91 (0.97–36.09)**	**0.05**
Not lifting weights	0.41 (0.69–2.53)	0.344
**Age**	**0.90 (0.84–0.97)**	**0.007**
Sex	1.89 (0.16–21.42)	0.607
Disease subset at diagnosis	1.95 (0.28–13.51)	0.49
Lack of DUs (ever)	0.34 (0.07–1.48)	0.153
Joint contractures	0.63 (0.11–3.58)	0.609
mRSS	0.99 (0.85–1.15)	0.935
SHAQ score	0.99 (0.97–1.00)	0.277

## Data Availability

The data that support the findings of this study are available upon reasonable request from the corresponding author, C.A.V.

## Abbreviations

The following abbreviations are used in this manuscript:

SSc	systemic sclerosis
ES	employment status
EUSTAR	The European Scleroderma Trials and Research Group
DU(s)	digital ulcer(s)
OR	odds ratio
CI	Confidence Interval ADL—activities of daily life
AS	ankylosing spondylitis
RA	rheumatoid arthritis
ER	early retirement
lSSc	limited cutaneous systemic sclerosis
dSSc	diffuse cutaneous systemic sclerosis
mRSS	modified Rodnan skin score
SHAQ	Scleroderma Health Assessment Questionnaire
DHI	Duruoz Hand Index
VAS	Visual Analog Scale
IBM SPSS	International Business Machines Corporation—Statistical Package for the Social Sciences
FVC	forced vital capacity
